# Clinical utility of right atrial strain to estimate pulmonary hypertension in comparison with right ventricular free wall longitudinal strain

**DOI:** 10.20407/fmj.2024-022

**Published:** 2024-12-27

**Authors:** Shinji Jinno, Akira Yamada, Maho Kawashima, Hideo Izawa

**Affiliations:** 1 Clinical Laboratory, Fujita Health University Hospital, Toyoake, Aichi, Japan; 2 Department of Cardiology, Fujita Health University, School of Medicine, Toyoake, Aichi, Japan

**Keywords:** Right atrial strain, Speckle-tracking, Pulmonary hypertension

## Abstract

**Objectives:**

This study aimed to measure right atrial (RA) strain in the reservoir, conduit, and contraction phases and examine its clinical utility in detecting pulmonary hypertension (PH).

**Methods:**

One hundred and thirteen patients hospitalized in the intensive or coronary care units of our institution who underwent echocardiography and measurements of RA/right ventricular (RV) strain were retrospectively examined. RA strain was measured in the reservoir, conduit, and contraction phases of one cardiac cycle. PH was defined as peak tricuspid regurgitation velocity >2.8 m/s. Patients were grouped according to PH status (PH, no PH) and statistically compared. Logistic regression and receiver operating characteristic analyses were also performed.

**Results:**

Mean age was 71.1±15.4 years and 72 were men (63.7%). The PH and no PH groups comprised 40 and 73 patients, respectively. Among the RA strain parameters, RA strain in the conduit phase was significantly lower in the PH group (–8.1±4.2% vs. –17.4±7.7%; p<0.001). In the receiver operating characteristic analysis for PH, RA strain in the conduit phase had the highest area under the curve among the RA/RV strain parameters (area under the curve, 0.88; sensitivity, 92.5%; specificity, 71.2%; p<0.001).

**Conclusions:**

RA strain is an echocardiographic parameter that can detect PH and should be considered when RV strain parameters are not measurable.

## Introduction

Early diagnosis of pulmonary hypertension (PH) has become clinically important owing to recent advances in its treatment. Although invasive catheterization is required to definitively diagnose PH, echocardiography plays a role in screening and is more practical for clinical follow-up because of its noninvasive nature.

Regardless of underlying etiology, PH causes right ventricular (RV) pressure overload and dysfunction, which can be detected on echocardiography. Provided that pulmonary stenosis is not present, systolic pulmonary arterial pressure (PAP) can be estimated using echocardiographic parameters and the Bernoulli equation. This equation is first used to calculate the peak tricuspid regurgitation (TR) pressure gradient from measured TR velocity (TRV). Then, estimated right atrium pressure (RAP) is added to the calculated pressure gradient to determine systolic PAP.^1^ However, this method can be problematic because of the squaring function in the Bernoulli equation and the possibility of an inaccurately estimated RAP.^2^ Therefore, the latest guidelines for PH screening recommend using peak TRV value rather than estimated systolic PAP.^1–3^ However, echocardiographic TRV measurements can be lower than the actual values when imaging of the TR jets is not clear. In addition, eccentric TR jets can be difficult to accurately evaluate owing to an inappropriate Doppler angle.^4^ Furthermore, measurement errors may occur when the envelope of a distinct TR waveform is not properly delineated.^5^

RV free wall longitudinal strain (RVFWSL) obtained using two-dimensional (2D) speckle tracking echocardiography (STE) has been acknowledged as a reliable index of RV function. RVFWSL is correlated with mean PAP as measured via right heart catheterization.^6,7^ However, it is not always possible to measure RVFWSL, such as when the free wall of the RV cannot be clearly imaged in patients with advanced pulmonary disease.^8,9^

RA strain is a prognostic factor in various cardiac diseases and can be used to estimate RAP.^10,11^ Moreover, it can be measured using 2D STE in the reservoir, conduit, and contraction phases. Most previous RA strain studies have focused on RA reservoir strain. This study aimed to measure RA strain in all three phases and examine its clinical utility in detecting PH.

## Methods

### Study patients

All patients admitted to the intensive or coronary care units at Fujita Health University Hospital, Toyoake, Japan between October 2020 and February 2022 who underwent echocardiography on the first day of admission were retrospectively reviewed. Those in whom both RA strain and RVFWSL were measurable and had clear TR jet waveforms suitable for TRV measurement were eligible for study inclusion. We excluded patients with non-sinus rhythm, acute right ventricular infarction, and acute pulmonary thromboembolism. Those on mechanical ventilation and patients whose echocardiographic imaging quality was poor were also excluded.

### Echocardiography

Conventional 2D echocardiography was performed using a Vivid E95 system (GE Healthcare, Chicago, IL, USA) with an M5Sc-D transducer (1.4–4.6 MHz). Cardiac dimensions and volumes and Doppler parameters were measured according to the recommendations of the American Society of Echocardiography.^8,12^ All images were obtained at a frame rate of 60 to 80 frames/sec.

Three consecutive cardiac cycles were saved in digital format and the one most suitable for strain analysis was selected. RA and RV strain were measured according to the European Association of Cardiovascular Imaging standards.^13^ RA strain was analyzed using an RV-focused apical four-chamber view with optimized orientation, depth. and gain to maximize RA area without RA foreshortening to visualize the entire RA throughout a cardiac cycle. For RA strain analysis, the R wave on electrocardiography was used as the reference. RA strain was measured during the reservoir phase (RASr), conduit phase (RAScd), and contraction phase (RASct). RVFWSL measurements excluded the interventricular septum for strain analysis. After adjusting the region of interest to include the entire myocardial layer, the tracking quality was validated throughout the cardiac cycle.

An examiner unaware of the TRV values performed strain analysis offline using EchoPAC PC software version 204 (GE Healthcare). To examine intra- and interrater reproducibility of strain measurements, both RA strain and RVFWSL were recalculated in 27 patients by the same examiner and by another examiner, respectively. TR jets were recorded in multiple views: the waveform with the highest TRV was used. TRV >2.8 m/sec was defined as PH.^1^ Patients were classified according to PH status (PH or no PH).

### Statistical analysis

Statistical analyses were performed using JMP Pro software version 17.2.0 (SAS Institute, Cary, NC, USA). Two-sided p<0.05 was considered significant. Data are presented as mean±standard deviation or as frequency (percentage). The Shapiro–Wilk test was used to assess the normality of continuous data. Normally distributed data were compared using the Student’s unpaired t-test; the Wilcoxon signed rank test was used to compare non-normally distributed data. Categorical variables were compared using the chi-square test or Fisher’s exact test, as appropriate. Receiver operating characteristic (ROC) curve analysis was used to identify parameters with the best performance for detecting PH by determining the area under the ROC curve (AUC). Factors significantly associated with PH in univariate nominal logistic regression analyses (p<0.05) were analyzed using a multivariate model to identify factors independently associated with PH. Intra- and interobserver reliability was analyzed using the intraclass correlation coefficient.

### Ethics statements

The study was conducted in accordance with the principles of the Declaration of Helsinki. Institutional ethics committee approval was obtained (approval number, HM20-161). The requirement for informed consent was waived, owing to the retrospective nature of the study.

## Results

### Intra- and interobserver reliability

Intra- and interobserver reliability was high for measurements of RA strain in all three phases and RVFWSL. The intraclass correlation coefficients for intra-and interobserver reliability for RASr were 0.98 and 0.96, respectively. Corresponding values for RAScd were 0.97 and 0.97, respectively. Corresponding values for RASct were 0.97 and 0.98, respectively. Values for RVFWSL were 0.99 and 0.98, respectively.

### Patient characteristics

Two hundred thirty-one patients were eligible for study inclusion based on the criteria. RA strain and RVFWSL were measurable in 222 (96%) and 156 (68%) patients, respectively (p<0.001). One hundred thirteen patients (mean age, 71.1±15.4 years; 72 men) had measurable RA strain and RVFWSL, as well as measurable TR waveforms, and were included for analysis. The PH and no PH groups comprised 40 and 73 patients, respectively. Patient characteristics according to group are shown in Table 1. Mean age was significantly higher (74.8±12.5 years vs. 69.1±16.5 years; p<0.001) and mean body weight (54±12 kg vs. 60±13 kg; p=0.02) and height (157±9 cm vs. 161±10 cm; p=0.02) were significantly lower in the PH group. Body mass index did not significantly differ between the groups. The prevalence of chronic kidney disease (90% vs. 58%; p<0.001) was significantly higher, and the prevalence of diabetes (8% vs. 29%; p=0.005) was significantly lower in the PH group. Other clinical parameters did not significantly differ between the groups.

### Echocardiographic data

Table 2 shows 2D and Doppler echocardiographic data. Left ventricular (LV) end-systolic volume was significantly higher (96.3±50.2 mL vs. 65.5±32.7 mL; p=0.001), and LV ejection fraction (LVEF) was significantly lower (40.8±13.2% vs. 49.0±11.3%; p=0.002) in the PH group. Echocardiographic parameters of RV systolic function (tricuspid annular plane systolic excursion [15.4±4.6 mm vs. 17.5±4.7 mm; p=0.023], S' [9.7±3.4 cm/s vs. 11.0±3.1 cm/s; p=0.041], and RV fractional area change [FAC] [32.9±8.6% vs. 37.7±6.8%; p=0.003] were significantly lower in the PH group. The proportion of patients with moderate or severe TR was significantly higher in the PH group (18% vs. 6%; p=0.045). However, RA area did not significantly differ between the PH and no PH groups (16.8±4.2 cm^2^ and 15.6±3.5 cm^2^, respectively; p=0.135).

RV and RA strain parameters derived from 2D STE are shown in Table 3. RVFWSL (–20.4±7.3% vs. –25.1±5.0%; p=0.001), RASr (24.4±8.2% vs. 32.4±11.2%; p<0.001) and RAScd (−8.1±4.2% vs. −17.4±7.7%; p<0.001) were significantly lower in the PH group. RASct did not significantly differ between the PH and no PH groups (−16.2±7.5% and −14.9±7.1%, respectively; p=0.384).

### ROC analysis

**Figure 1:**
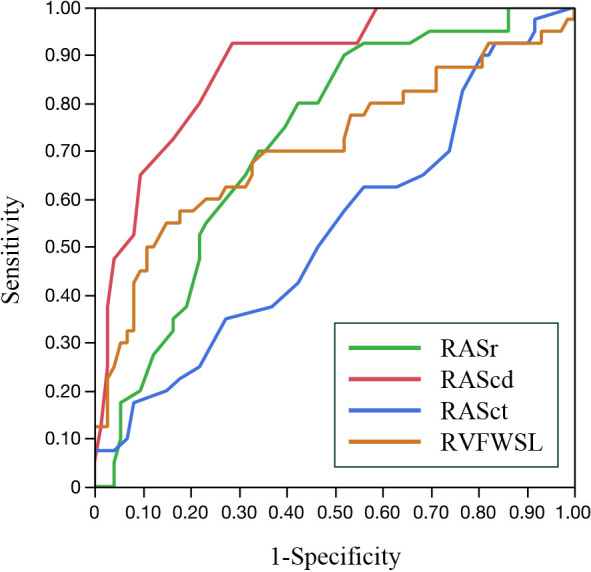
ROC curve analysis for the detection of PH ROC, receiver operating characteristic; PH, pulmonary hypertension; RA, right atrial; RASr, RA strain during reservoir phase; RAScd, RA strain during conduit phase; RASct, RA strain during contraction phase; RV, right ventricular; FWSL, free wall longitudinal strain.

The results of the ROC analysis for detection of PH are shown in Figure 1 and Table 4. Among the 2D STE right heart parameters, RAScd had the largest AUC (AUC, 0.88; sensitivity, 92.5%; specificity, 71.2%), followed by RASr (AUC, 0.72; sensitivity, 90.0%; specificity, 48.0%) and RVFWSL (AUC, 0.71; sensitivity, 55.0%; specificity, 84.9%). The AUC for RAScd was significantly higher than the AUC for RVFWSL (p=0.003), RASr (p<0.001), and RASct (p<0.001).

### Univariate and multivariate analyses of associations between RA strain and PH

In the univariate logistic regression analyses, LV end-diastolic volume, LV end-systolic volume, LVEF, LV mass index, relative wall thickness, left atrial volume index, E/e', RV FAC, tricuspid annular plane systolic excursion, S', and moderate or severe TR were significantly associated with PH. Multivariate analysis adjusted for age, sex, body mass index, LVEF, E/e', RV FAC, and moderate or severe TR demonstrated that RAScd was independently associated with PH (β=0.28; 95% confidence interval [CI], 0.16–0.44; p<0.001).

## Discussion

The results of this study showed that measurement of RA strain is feasible for detecting PH. RA strain was measurable in a significantly higher proportion of patients than RVFWSL was. RASr, RAScd, and RVFWSL were significantly lower in patients with PH. Although these three strain indices exhibited acceptable diagnostic performance for detecting PH, RAScd had the best performance and was independently associated with PH, according to multivariate analysis. These results suggest that RAScd would be useful as a noninvasive indicator for diagnosing and clinically monitoring PH over time.

Both RA strain and RV strain are analyzed in the RV-focused apical four-chamber view. The RV is susceptible to lung artifacts and can be difficult to scan in some cases. Even slightly unclear RV free wall images at any point in the cardiac cycle can cause deterioration in tracking accuracy. RA strain analysis was not possible in only a small number of our patients because of pericardial or pleural effusion or the presence of catheters, leads, or other foreign objects. In a study of patients with precapillary PH (pPH), RA strain and RV strain was measurable in 93% and 88% of patients, respectively, indicating that RA strain would be a practical index to use in clinical practice.^11^

Assessment of RA phasic functional parameters is clinically important, because the RA is a dynamic structure involved with RV filling. The three components of RA function are: (1) reservoir function, storing blood during tricuspid closure; (2) conduit function, passive blood transfer directly from the coronary and systemic veins to the RV during tricuspid opening; and (3) booster pump function, atrial contraction in late diastole to complete ventricular filling.^14^

In the initial stage of pulmonary pressure increase, RV diastolic dysfunction occurs to some extent, despite preserved RV systolic function. This alteration impacts RA performance to a detectable extent. As PH advances, the alteration in RA function becomes explicit, that is, the reservoir and conduit period reflected by positive RA strain decreases; however, the contraction period or negative strain increases, compensating the RA ejection volume.^4^

According to a cardiac magnetic resonance study of patients with pPH and healthy controls, pPH patients have impaired RA strain.^15^ More importantly, even in pPH patients with preserved RV systolic function, changes in RA and RV strain were observed (lower RAScd, in particular). In another cardiac magnetic resonance study,^16^ multivariate analysis showed that RAScd was independently associated with worse cardiovascular outcomes in patients with heart failure with preserved ejection fraction in sinus rhythm; the study also showed that impaired RAScd was significantly associated with lower N-terminal prohormone of brain natriuretic peptide concentration, systolic PAP, and pulmonary vascular resistance.

Our results are consistent with these studies, as we also found that RAScd is significantly associated with PH and systolic PAP. Another previous study of treatment-naïve patients with PH reported a significant improvement in RAScd after treatment; however, RASr and RASct remained unchanged.^17^

According to two meta-analyses that examined the normal range of RA strain,^18,19^ the RAScd cutoff values vary from 18% (95% CI, 7%–28%) to 23.6% (95% CI, 20.7%–26.6%). Such a wide reference range may limit the utility of RA strain in clinical practice. Nevertheless, in our study, –12.0% was the optimal RAScd cutoff for detecting PH (TRV >2.8 m/s), which is considered reasonable in comparison with the normal cutoff values, as mentioned above. We also showed that RA strain was associated with TRV. Establishing reference values for RA strain would require future large-scale multicenter studies that implement invasive testing. Nonetheless, RA strain is practical to use and highly reproducible. RAScd should be measured for estimating PH, especially in emergency admissions.

## Conclusions

RA strain is an echocardiographic parameter that can detect PH. RAScd could be a surrogate marker that reflects increased TRV. RA strain should be measured, especially when TRV and/or RV strain are difficult to measure.

## Figures and Tables

**Table1:** Patient characteristics

	PH group (n=40)	no PH group (n=73)	p-value
Men, n (%)	26 (65)	46 (63)	0.834
Age (years)	75±12	69±16	0.040
Weight (kg)	54±12	60±13	0.015
Height (cm)	157±9	161±10	0.024
Body mass index (kg/m^2^)	21.5±3.8	22.8±3.7	0.097
Risk factors			
Diabetes, n (%)	2 (8)	21 (29)	0.005
Hypertension, n (%)	14 (35)	27 (37)	0.834
Dyslipidemia, n (%)	7 (18)	5 (7)	0.086
Chronic kidney disease, n (%)	36 (90)	42 (58)	<0.001
Reason for hospitalization			0.010
Acute heart failure, n (%)	21 (53)	20 (27)	
Acute coronary syndrome, n (%)	10 (25)	32 (44)	
Acute myocarditis, n (%)	0 (0)	5 (7)	
Non cardiac disease, n (%)	9 (23)	16 (22)	
Systolic blood pressure (mm Hg)	121±30	126±23	0.412
Diastolic blood pressure (mm Hg)	72±24	70±17	0.630
Heart rate (bpm)	78±13	76±14	0.485

Data are expressed as numbers (percentage) or means±standard deviation.

**Table2:** Two-dimensional and Doppler echocardiographic parameters

	PH group (n=40)	no PH group (n=73)	p-value
LVEDV (mL)	130.9±66.7	108.1±42.8	0.058
LVESV (mL)	96.3±50.2	65.5±32.7	0.001
LVEF (%)	40.8±13.2	49.0±11.3	0.002
LAVI (mL/m^2^)	51.5±24.1	38.6±16.8	0.005
LVMI (g/m^2^)	144.8±41.6	121.7±36.5	0.005
RWT	0.38±0.10	0.42±0.09	0.029
Average E/e'	18.3±9.7	13.0±6.4	0.003
TAPSE (mm)	15.4±4.6	17.5±4.7	0.023
S' (cm/sec)	9.7±3.4	11.0±3.1	0.041
RV FAC (%)	32.9±8.6	37.7±6.8	0.003
RV Tei index	0.57±0.41	0.41±0.34	0.073
RV E/A	0.9±0.3	1.0±0.4	0.069
RV E DT	135.1±62.3	151.3±64.8	0.274
RA area (cm^2^)	16.8±4.2	15.6±3.5	0.135
Moderate or severe TR	7 (18)	4 (6)	0.045
TRV (m/sec)	3.3±0.4	2.3±0.3	<0.001
RAP grades 3/8/15 mm Hg	31/6/3	57/12/4	0.903

LVEDV, left ventricular end-diastolic volume; LVESV, left ventricular end-systolic volume; LVEF, left ventricular ejection fraction; LAVI, left atrial volume index; LVMI, left ventricular mass index; RWT, relative wall thickness; TAPSE, tricuspid annular plane systolic excursion; RV, right ventricular; FAC, fractional area change; DT, deceleration time; RA, right atrial; TR, tricuspid regurgitation; TRV, peak tricuspid regurgitation velocity; RAP, right atrial pressure Data are expressed as means±standard deviation or numbers (percentage).

**Table3:** Two-dimensional speckle tracking echocardiographic parameters

	PH group (n=40)	no PH group (n=73)	p-value
RVFWSL (%)	–20.4±7.3	–25.1±5.0	0.001
RASr (%)	24.4±8.2	32.4±11.2	<0.001
RAScd (%)	–8.1±4.2	–17.4±7.7	<0.001
RASct (%)	–16.2±7.5	–14.9±7.1	0.384

RVFWSL, right ventricular free wall longitudinal strain; RASr, right atrial strain during reservoir phase; RAScd, right atrial strain during conduit phase; RASct, right atrial strain during contraction phase Data are expressed as means±standard deviation.

**Table4:** Receiver operating characteristic curve analysis of clinical and echocardiographic variables for detecting pulmonary hypertension

	AUC	p-value	Cut-off value (%)	Sensitivity (%)	Specificity (%)
RVFWSL	0.71	<0.001	–19.9	55.5	84.9
RASr	0.72	<0.001	33.0	90.0	48.0
RAScd	0.88	<0.001	–12.0	92.5	71.2
RASct	0.54	0.371	–24.0	17.5	91.8

AUC, area under the receiver operating characteristic curve; RVFWSL, right ventricular free wall longitudinal strain; RA, right atrial; RASr, right atrial strain during reservoir phase; RAScd, right atrial strain during conduit phase; RASct, right atrial strain during contraction phase
